# Clinical characteristics and survival of glioblastoma complicated with non-central nervous system tumors

**DOI:** 10.1186/s41016-022-00312-1

**Published:** 2022-12-27

**Authors:** Chen Wang, Di Wang, Changqing Pan, Jiazheng Zhang, Cheng Cheng, You Zhai, Mingchen Yu, Zhiliang Wang, Guanzhang Li, Wei Zhang

**Affiliations:** 1 Department of Neurosurgery, Beijing Tiantan Hospital, Capital Medical University, No. 119 South Fourth Ring Western Road, Fengtai District, Beijing, China; 2 Beijing Neurosurgical Institute, Capital Medical University, No. 119 South Fourth Ring Western Road, Fengtai District, Beijing, China; 3 Center of Brain Tumor, Beijing Institute for Brain Disorders, Beijing, China; 4 China National Clinical Research Center for Neurological Diseases, Beijing, China; 5 Chinese Glioma Genome Atlas (CGGA), Beijing, China

**Keywords:** Glioblastoma, Multiple primary tumors, Clinical and pathological characteristics, Prognosis

## Abstract

**Background:**

Diagnosis and treatment of patients with glioblastoma (GBM) who are also diagnosed with primary non-central nervous system (CNS) tumors remain a challenge, yet little is known about the clinical characteristics and prognosis of these patients. The data presented here compared the clinical and pathological features between glioblastoma patients with or without primary non-CNS tumors, trying to further explore this complex situation.

**Methods:**

Statistical analysis was based on the clinical and pathological data of 45 patients who were diagnosed with isocitrate dehydrogenase (IDH) wild-type glioblastoma accompanied by non-CNS tumors between January 2019 and February 2022 in Beijing Tiantan Hospital. Univariate COX proportional hazard regression model was used to determine risk factors for overall survival.

**Results:**

It turned out to be no significant difference in the overall survival (OS) of the 45 patients with IDH-wild-type GBM plus non-CNS tumors, compared with the 112 patients who were only diagnosed with IDH-wild-type GBM. However, there was a significant difference in OS of GBM patients with benign tumors compared to those with malignant tumors.

**Conclusions:**

Implications for the non-central nervous system tumors on survival of glioblastomas were not found in this research. However, glioblastomas complicated with other malignant tumors still showed worse clinical outcomes.

## Background

Glioblastoma complicated with other non-CNS neoplasms is a challenging clinical problem, and the managing clinical risks of which has not been fully explored. Several case series observed an increasing part of GBM patients have been diagnosed with non-CNS tumors previously, which were classified as multiple primary malignant neoplasm (MPMN) [1]. Previous studies showed that the patients survived from cancer are at increased risk of developing the second or even the third primary tumors [2, 3]. On the other hand, it remains inconclusive whether other neoplasms affect the prognosis of patients with primary isocitrate dehydrogenase (IDH) wild-type glioblastoma. Considering such an inconclusive factor may influence the accuracy of clinical trials and cohort studies of gliomas, and it is important to uncover the clinical and pathological characteristics of these patients. In this study, we retrospectively investigated the clinical and molecular pathological characteristics of these patients based on the glioblastoma patient cohort from Beijing Tiantan Hospital and the Chinese Glioma Genome Atlas (CGGA).

## Methods

### Patients

Clinical data of 117 patients who were diagnosed with primary glioblastoma plus non-CNS tumors were collected from Beijing Tiantan Hospital from January 2019 to February 2022. According to the fifth edition of the WHO classification of tumors of the central nervous system, 45 patients who were pathologically diagnosed with IDH-wild-type were finally enrolled in this study [4]. And the data of patients diagnosed with GBM only was collected from CGGA 325 database as the matched group.

### Collection of data

The specific data included age at diagnosis of GBM, gender, date of receiving surgical operation of GBM, date of death or the last following-up, O6-methylguanine-DNA methyltransferase (MGMT) promoter status, telomerase reverse transcriptase (TERT) promoter status, and postoperative radiotherapy and chemotherapy status. The following-up ended on April 9, 2022. For comparison, the paralleled data of the patients who were diagnosed with GBM only between January 2019 and February 2022 was collected from the CGGA database. The primary endpoint was OS, defined as the time interval between the day of surgical operation for GBM and the patient’s death or last following-up.

### Statistical analysis

All statistical analyses were conducted with IBM SPSS 25. For normally distributed data or non-normally distributed data, it will be expressed as the mean ± SD or median, respectively. For the two groups, Pearson’s chi-squared (*χ*^2^) test was employed to analyze the categorical variables. And student *t* test and Mann–Whitney *U* test were utilized to evaluate the continuous variables. Survival analyses were performed through the Kaplan–Meier method and the differences in survival rates between the two groups were compared by using the log-rank test. Univariate COX proportional hazard regression model analyses were applied to determine the factors that affect the OS. A *P* value < 0.05 was considered statistically significant.

## Results

### General characteristics of patients

**Fig. 1:**
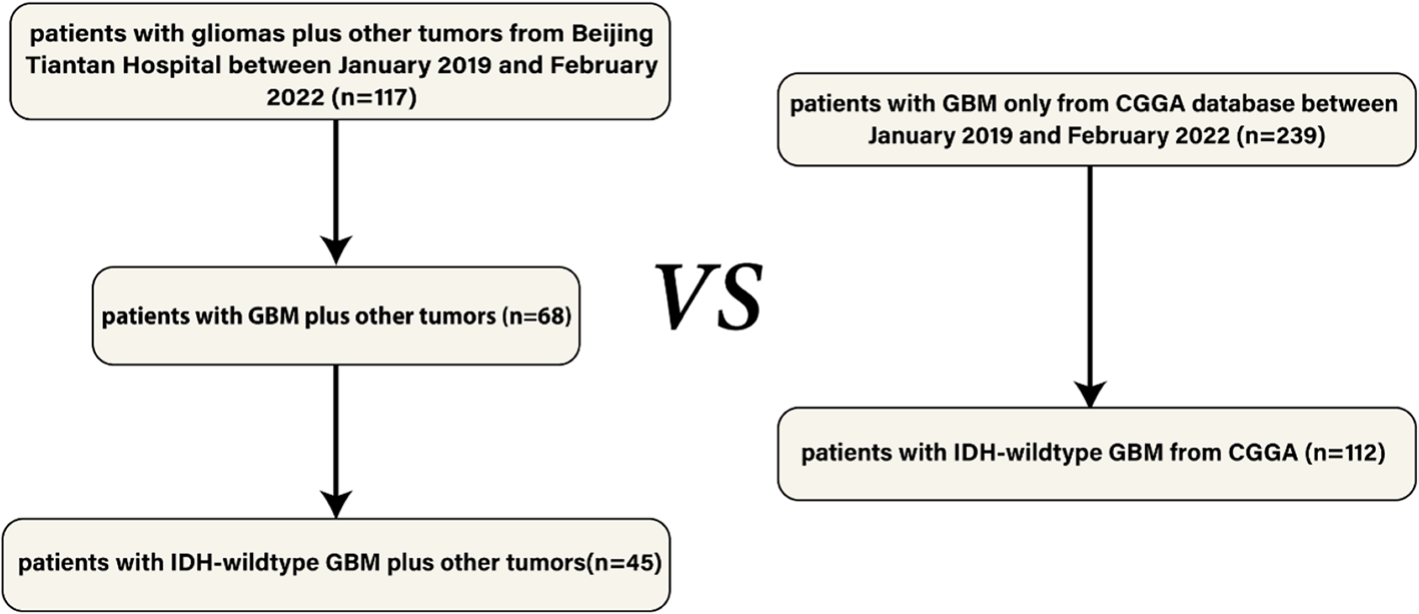
Flowchart of the enrollment of the glioma patients in the two groups. GBM, glioblastoma

For the group of GBM patients plus primary tumors of other sites, 45 cases were finally enrolled. And for the group of patients diagnosed with GBM only, 112 cases that met the criteria of selection were picked (Fig. 1).

### Distribution of non-CNS tumors in the patients

Based on our study, it seemed that female patients are more likely to develop multiple primary tumors (*n* = 35, 77.8%). And the most common non-CNS primary tumor was the hysteromyoma of the female patients (*n* = 17, 37.8%). Meanwhile, the second common no-CNS tumor was the breast tumor (*n* = 8, 17.8%), including breast cancer (*n* = 5) and breast adenoma (*n* = 3). For the male patients, urinary system tumors were the most common (*n* = 4, 8.9%), including bladder tumors (*n* = 3) and clear cell renal carcinoma (*n* = 1). When classified according to the system, reproductive system tumors of the female were the most part in our study (*n* = 30, 66.7%), which was comprised of hysteromyoma (*n* = 17), breast tumors (*n* = 8), endometrial carcinoma (*n* = 3), and ovarian tumors (*n* = 2). Urinary system tumors were the second most common (*n* = 6, 13.3%), which were composed of renal tumors (*n* = 3) and bladder tumors (*n* = 3). And the incidence of the digestive system tumors was the next (*n* = 4, 8.9%), including colorectal cancer (*n* = 2), gastric cancer (*n* = 1), and ampulla carcinoma (*n* = 1). The rest multiple primary tumors included thyroid cancer (*n* = 3), based cell carcinoma (*n* = 2), lumbar tumor (*n* = 1), and nasal lymphosarcoma (*n* = 1). Moreover, there were three GBM patients diagnosed with two non-CNS primary tumors.

### Comparison of clinical characteristics and survival trends between two groups

As exhibited in Table 1, the group of GBM patients with non-CNS tumors got an older age at diagnosis for GBM when compared to the group of patients with GBM only (median age 56.00 compared to 53.50, *p* = 0.002). And for the aspect of gender, it seems that the female patients diagnosed with GBM tend to have more possibilities to develop multiple primary tumors than the male (77.80% of female patients in the group of GBM plus non-CNS tumors compared to 36.60% of female patients in the group of GBM only, *p* < 0.0001). For the mutation status of the TERT promoter, the group of GBM patients with non-CNS tumors consisted of a higher proportion of the status of TERT promoter mutation than the other group (68.40% TERT promoter mutation status in the former group compared to 47.00% in the latter group, *p* = 0.034). However, there was no significant difference in the status of MGMT promoter methylation status between the two groups (50% MGMT promoter methylation in the 34 patients from the group of GBM plus non-CNS tumors compared to 38.2% in the 68 patients from the group of GBM only, *p* = 0.257). More importantly, no statistically significant difference in OS was observed between the two groups (79.47 of mean rank for the group of GBM plus non-CNS tumors compared to 69.33 for the other group, *p* = 0.177).

**Table 1:** Clinical and pathological characteristics of the enrolled GBM patients at baseline

Characteristic	GBM only	GBM plus non-CNS tumor	*P* value
Age (years)			
Median	53.50(34–84)	56.00(12–76)	0.002
Gender			
Total	112	45	0.000
Female	41 36.6%	35 77.8%	
Male	71 63.4%	10 22.2%	
OS (days)			
Total	99	45	0.177
Mean rank	69.33	79.47	
TERT status			
Total	66	38	0.034
Mutant	31 47.00%	26 68.40%	
Wild type	35 53.00%	12 31.60%	
MGMT promoter status			
Total	68	34	0.257
Methylation	26 38.20%	17 50.00%	
Non-methylation	42 61.80%	17 50.00%	
Censor			
Total	108	45	0.946
Alive	75 69.40%	31 68.90%	
Dead	33 30.60%	14 31.10%	

*GBM* Glioblastoma, *CNS* Central nervous system, *OS* Overall survival, *TERT* Telomerase reverse transcriptase, *MGMT* O6-methylguanine-DNA methyltransferase

**Fig. 2:**
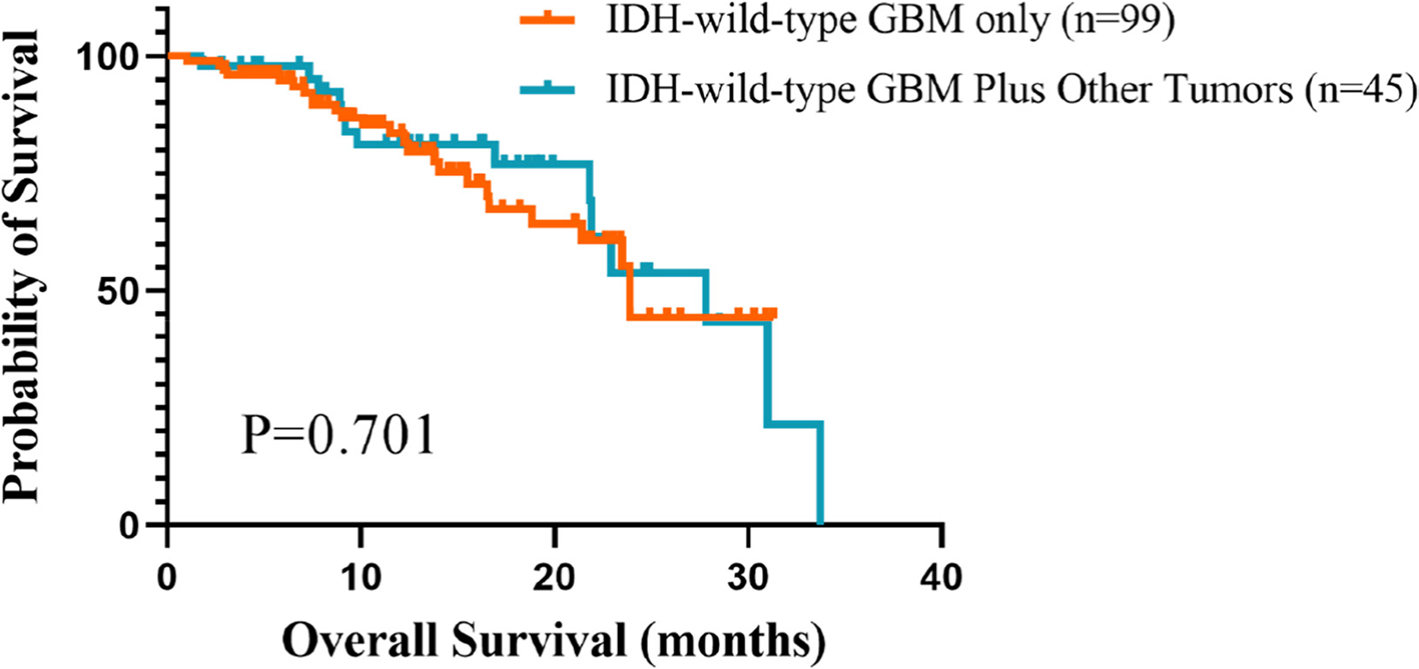
Kaplan–Meier estimates of overall survival (OS) time for patients with IDH-wild-type GBM only and patients with IDH-wild-type GBM plus other tumors. Note: In the group of IDH-wild-type GBM patients, the overall survival data of 13 patients are not available. Hence, there are 99 patients left in the end. Note: GBM glioblastoma

**Fig. 3:**
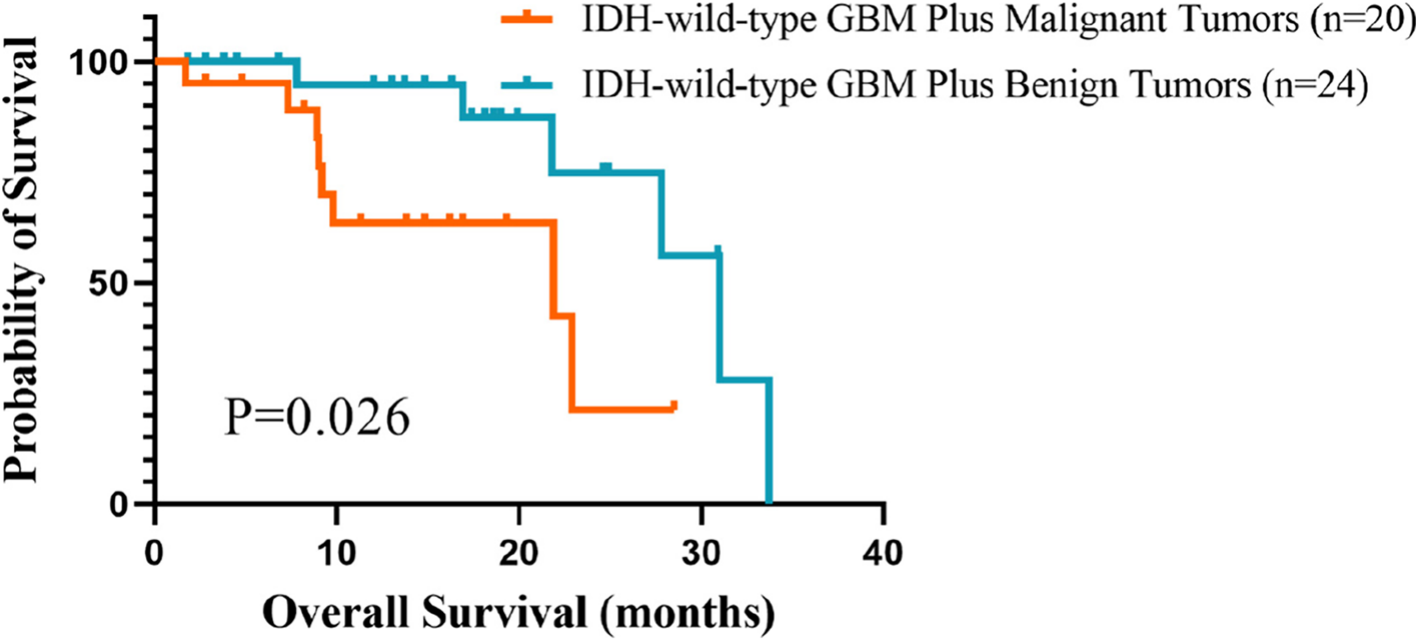
Kaplan–Meier estimates of overall survival (OS) time for IDH-wild-type GBM patients with benign tumors and malignant tumors. Note: For this group of 45 GBM patients with other tumors, it is hard to tell the malignancy of the complicated tumor of one patient according to the medical record. Therefore, there are 44 patients in all left in this group. Note: GBM glioblastoma

For the analyses of survival, there was no significant difference in the comparison of OS between the group of GBM patients with non-CNS tumors and patients with GBM only (median OS 27.8 months for the former group compared to 23.9 months for the latter, *p* = 0.701) (Fig. 2). Considering that the primary non-CNS tumors include the benign or malignant, which could be an important factor affecting the prognosis, we conducted a further comparison on the OS in the group of GBM patients with non-CNS tumors. And it was observed that there was significant difference in OS between the two groups (median OS 32.06 months for GBM patients with primary benign tumors and 22.83 months for GBM patients with primary malignant tumors, *p* = 0.026) (Fig. 3).

### Univariable analysis of prognostic factors

Univariable Cox proportional hazard regression analysis revealed that the status of postoperative adjuvant therapy was the only factor affecting the survival time of patients (*p* < 0.001). Furthermore, receiving the chemotherapy and receiving both chemotherapy and radiotherapy were the strongest prognostic factors for the OS of patients (*p* < 0.001), while receiving the radiotherapy alone meant little (Table 2).

**Table 2:** Univariate COX proportional hazard regression model analysis of factors associated with the survival of IDH-wild-type GBM patients

Covariate	B	SE	Hazard ratio	95%	CI	*P* value
Age	0.026	0.014	1.026	0.998	1.055	0.054
Gender	0.044	0.163	1.045	0.760	1.438	0.785
Multi-tumor						
GBM only vs GBM plus other tumors	0.138	0.346	1.148	0.583	2.260	0.689
TERT status						
TERT wild type vs TERT mutant	0.489	0.412	1.631	0.727	3.658	0.235
MGMT promoter status						
MGMT Un-methy vs MGMT Methy	0.173	0.388	1.189	0.556	2.542	0.656
Postoperative adjuvant therapy						0.000
Radiotherapy vs both	0.271	0.741	1.312	0.307	5.600	0.714
Chemotherapy vs Both	3.019	0.806	20.463	4.220	99.231	0.000
Neither vs both	2.583	0.445	13.238	5.537	31.650	0.000

*B* partial regression coefficient, *SE* Standard error, *95% CI* 95% Confidence interval, *GBM* Glioblastoma, *Methy* Methylation, *postoperative adjuvant therapy* radiotherapy and chemotherapy

## Discussion

The first research concerning the multiple primary tumors could trace back to 1921, reporting the incidence of 4.7% in 3000 patients diagnosed with malignant tumors [5]. Continued studies were conducted to find out the possible connections and impact [6, 7]. And the results tend to be similar and limited in various cancers [8, 9]. Almost no significant difference was observed when the survival time of patients with multiple tumors was compared to the patients with a single tumor in the past studies [10–12].

In recent years, with the great progress of diagnosis and treatment for tumors, the overall survival of cancer patients has been positively changed, which, however, led to the increased number of patients with multiple primary tumors [7, 13]. A similar phenomenon in GBM patients attracted our attention that usually led to a confusing situation for doctors to develop management plans for them or for researchers to recruit them into clinical trials. Meanwhile, there were few studies on GBM patients with multiple primary tumors in the past 5 years. And the fifth WHO guideline for the classification of CNS tumors identified the GBM as the IDH-wild-type, revolutionizing the understanding and clinical practice. It is worth conducting the study under this new situation.

In the beginning, we collect the data of all patients diagnosed with gliomas and other primary tumors, trying to figure out the difference of clinical and pathological characteristics and the prognosis compared to the control group which consisted of patients with gliomas only. From January 2019 to February 2022, 117 glioma patients were diagnosed with multiple primary tumors. However, considering the relatively better prognosis of patients with WHO II and WHO III gliomas, we finally determined to pick the 45 patients who were diagnosed with IDH-wildtype GBM to conduct the analyses.

We chose the GBM patients diagnosed with IDH-wild-type in the past 3 years, trying to reveal the clinical outcome and characteristics of the GBM patients with multiple primary tumors in the current situation to provide some possible guide for clinical practice. It was observed that the patients with multiple tumors usually got older age when they were diagnosed with GBM. And female patients who were frequently diagnosed with tumors of the reproductive system accounted for a large part in the special group based on our data. The distribution of non-CNS tumors was different from the past studies [14]. Since TERT promoter status and MGMT promoter status had been identified as the factors that strongly influence the prognosis of GBM patients, we choose them as the target to conduct the analysis of pathological characteristics [15–17]. Patients with multiple tumors tend to get the mutated TERT promoter, which implies that they may get a worse clinical prognosis [18]. And there was no significant difference in the MGMT promoter status between the two groups. For the last part, the postoperative adjuvant therapy was the only factor that affects the prognosis which was consistent with relevant clinical studies [19, 20].

For the analysis of overall survival, there was no significant difference between the two groups, which was consistent with the past studies [11, 14]. And the further analysis of OS between the GBM patients with benign tumors and GBM patients with malignant tumors showed significant difference, which meant that patients with benign tumors tend to have a longer median OS. According to our following-up results, GBM recurrence or progression is the main cause of death for the patients with primary non-CNS tumors. The malignant tumors diagnosed before, we presume, may have made an impact on the patients’ survival status.

For the limitation of our study, the lower number of patients may negatively and largely affect our analysis. And the number is possibly lower than the real due to the negligence in the process of history taking and recording. Meanwhile, limited data on molecular pathology could not support us to do further analysis on the possible connection between the multiple tumors. In addition, based on the history recording, the information about the complicated tumors is limited. Further exploration could not be conducted. How much of a role dose the complicated tumor play in the clinical outcome remains a question to this study.

To sum up, it is unexpected to find out that there was no significant difference in OS between the two groups. Based on our results, patients with malignant tumors before got a poorer survival outcome probably owing to the damage of the previous tumors or the relatively conservative treatment for them that could drive the progression or recurrence of GBM. Further studies including more patients and data on molecular pathology are urgently required to be conducted to better understand the clinical and pathological characteristics of this special group to provide more convincing guidance for clinical practice and trials. What is more, that may be able to discover some innate mechanism of multiple primary tumors and provide a support for the treatment.

## Data Availability

The datasets analyzed during the current study are available in CGGA database, http://www.cgga.org.cn/, and are available from the corresponding author on reasonable request. CGGA database (http://www.cgga.org.cn/) is a public database, and any researcher can download sequencing data and corresponding clinical data without login. Accession link to the database is http://www.cgga.org.cn/download.jsp. The DataSet ID is mRNAseq_325, Clinical Data. The molecular features, such as the status of IDH mutation, 1p/19q co-deletion status and MGMT promoter, etc., were collected in this dataset. Meanwhile, the patients’ follow-up information (histology, gender, age, WHO grade, overall survival and censor status, etc.) were also included in this dataset. All the subjects were diagnosed with gliomas by consensus, according to central pathology reviews by independent board-certified neuropathologists and further graded based on the 2007/2016 WHO classification. Written informed consent was obtained from all patients. The specimens were collected under IRB KY2013-017–01 and were frozen in liquid nitrogen within 5 min of resection.

## Abbreviations

GBM: Glioblastoma
CNS: Central nervous system
IDH: Isocitrate dehydrogenase
OS: Overall survival
MPMN: Multiple primary malignant neoplasm
CGGA: Chinese Glioma Genome Atlas
MGMT: O6-methylguanine-DNA methyltransferase
TERT: Telomerase reverse transcriptase

## References

[1] Vogt A, Schmid S, Heinimann K (2017). Multiple primary tumours: challenges and approaches, a review. ESMO open.

[2] Odani S, Tabuchi T, Nakata K (2022). Incidence and relative risk of metachronous second primary cancers for 16 cancer sites, Osaka, Japan, 2000–2015: population-based analysis. Cancer Med.

[3] Tanjak P, Suktitipat B, Vorasan N (2021). Risks and cancer associations of metachronous and synchronous multiple primary cancers: a 25-year retrospective study. BMC Cancer.

[4] Louis D, Perry A, Wesseling P (2021). The 2021 WHO classification of tumors of the central nervous system: a summary [J]. Neuro Oncol.

[5] Owen L (1921). Multiple malignant neoplasms. JAMA.

[6] Feller A, Matthes K, Bordoni A (2020). The relative risk of second primary cancers in Switzerland: a population-based retrospective cohort study. BMC Cancer.

[7] Donin N, Filson C, Drakaki A (2016). Risk of second primary malignancies among cancer survivors in the United States, 1992 through 2008. Cancer.

[8] Zhang L, Wu Y, Liu F (2016). Characteristics and survival of patients with metachronous or synchronous double primary malignancies: breast and thyroid cancer. Oncotarget.

[9] Chan G, Ong P, Low J (2018). Clinical genetic testing outcome with multi-gene panel in Asian patients with multiple primary cancers. Oncotarget.

[10] Amer MH (2014). Multiple neoplasms, single primaries, and patient survival. Cancer Manag Res.

[11] Hamza MA, Kamiya-Matsuoka C, Liu D (2016). Outcome of patients with malignant glioma and synchronous or metachronous non-central nervous system primary neoplasms. J Neurooncol.

[12] Wang H, Hou J, Zhang G (2019). Clinical characteristics and prognostic analysis of multiple primary malignant neoplasms in patients with lung cancer. Cancer Gene Ther.

[13] Coyte A, Morrison DS, McLoone P (2014). Second primary cancer risk-the impact of applying different definitions of multiple primaries: results from a retrospective population-based cancer registry study. BMC Cancer.

[14] Nguyen H, Doan N, Gelsomino M (2019). Management and survival trends for adult patients with malignant gliomas in the setting of multiple primary tumors: a population based analysis. J Neurooncol.

[15] Simon M, Hosen I, Gousias K (2015). TERT promoter mutations: a novel independent prognostic factor in primary glioblastomas. Neuro Oncol.

[16] Rivera A, Pelloski C, Gilbert M (2010). MGMT promoter methylation is predictive of response to radiotherapy and prognostic in the absence of adjuvant alkylating chemotherapy for glioblastoma. Neuro Oncol.

[17] Fujimoto K, Arita H, Satomi K (2021). TERT promoter mutation status is necessary and sufficient to diagnose IDH-wildtype diffuse astrocytic glioma with molecular features of glioblastoma. Acta Neuropathol.

[18] Stichel D, Ebrahimi A, Reuss D (2018). Distribution of EGFR amplification, combined chromosome 7 gain and chromosome 10 loss, and TERT promoter mutation in brain tumors and their potential for the reclassification of IDHwt astrocytoma to glioblastoma. Acta Neuropathol.

[19] Mirimanoff R, Gorlia T, Mason W (2006). Radiotherapy and temozolomide for newly diagnosed glioblastoma: recursive partitioning analysis of the EORTC 26981/22981-NCIC CE3 phase III randomized trial. J Clin Oncol.

[20] Stupp R, Dietrich P, OstermannKraljevic S (2002). Promising survival for patients with newly diagnosed glioblastoma multiforme treated with concomitant radiation plus temozolomide followed by adjuvant temozolomide. J Clin Oncol.

